# Effects of combined exercise training with sleep education in older adults with obstructive sleep apnea: protocol for a randomized clinical trial

**DOI:** 10.3389/fpsyg.2024.1322545

**Published:** 2024-02-15

**Authors:** Felipe Fank, Regiana Santos Artismo, Marcos Gonçalves de Santana, Andrea Maculano Esteves, Darlan Laurício Matte, Giovana Zarpellon Mazo

**Affiliations:** ^1^Laboratory of Gerontology, Health and Sports Sciences Center, Santa Catarina State University, Florianopolis, Brazil; ^2^Teaching, Research and Extension Center in Physiotherapy in the Pre- and Post-Operation of Major Surgeries, Health and Sports Sciences Center, Santa Catarina State University, Florianopolis, Brazil; ^3^Health Sciences Unit, Federal University of Jatai, Jatai, Brazil; ^4^Laboratory of Sleep and Exercise, School of Applied Sciences, State University of Campinas, Campinas, Brazil

**Keywords:** obstructive sleep apnea, combined training, aerobic exercise, sleep hygiene, strength training, older adults

## Abstract

**Background:**

Obstructive sleep apnea (OSA) is a common disorder that affects approximately 1 billion people worldwide. Advanced age is a significant risk factor. Various treatment options have been explored to reduce the severity of OSA symptoms and physical exercise has emerged as a potential alternative therapy. Therefore, this study aims to investigate the effects of a combined exercise program with sleep education on sleep quality and on the severity of OSA in older adults.

**Methods:**

This is a randomized clinical trial with two parallel groups that will involve individuals of both genders aged between 60 and 79 years who have an apnea-hypopnea index (AHI) of more than 15 events per hour and who have not received or are currently undergoing treatment for OSA. Older adults who have engaged in regular exercise in the last six months and individuals with contraindications to exercise will be excluded. The study will assess outcomes related to OSA, including AHI, oxygen desaturation index, minimum and mean oxyhemoglobin saturation, sleep efficiency, sleep latency, and the type of respiratory events. Additionally, sleep quality-related outcomes, daytime sleepiness, physical activity, physical fitness, aerobic capacity, cognitive status, anthropometric measures, and health-related quality of life will be analyzed. Participants will be randomized to two groups: a combined exercise group (involving both resistance and aerobic training) with sleep education, and a control group that will receive only educational recommendations for managing OSA. The intervention will last 12 weeks and will consist of three sessions per week, totaling 36 exercise sessions. Sample size calculation indicates a minimum number of 36 participants.

**Discussion:**

If the hypothesis is confirmed, this clinical trial will indicate an effective non-pharmacological intervention for treating OSA in older adults. This intervention could be used as an adjunct to existing approaches designed to improve OSA management.

**Clinical trail registration:**

Brazil Clinical Trials Registry (ReBEC), identifier RBR-9hk6pgz.

## 1 Introduction

Obstructive sleep apnea (OSA) is a highly prevalent breathing disorder that affects approximately 1 billion people worldwide (Benjafield et al., 2019). The prevalence of moderate to severe OSA ranges from 6 to 17% among adults and can reach up to 49% in older age groups (Senaratna et al., 2017). The main risk factors associated with OSA are obesity, male gender, craniofacial abnormalities, genetic predisposition, ethnic differences, and advanced age (Young et al., 2004; Jordan et al., 2014; Lee and Sundar, 2021).

Positive airway pressure therapy is the primary treatment for individuals with symptomatic OSA of any severity and continuous positive airway pressure (CPAP) is the gold standard among its modalities (Epstein et al., 2009; Gottlieb and Punjabi, 2020; Lee and Sundar, 2021). However, although CPAP is effective in many patients, it is not always well tolerated, a fact that can reduce patient adherence to treatment (Rotenberg et al., 2016). Additionally, CPAP can have some adverse effects (Sawyer et al., 2011), such as skin breakdown, conjunctivitis, nasal congestion, airway dryness, and gastrointestinal obstruction (Zandieh and Katz, 2010; Virk and Kotecha, 2016).

Recently, alternative treatments for OSA have been investigated and physical exercise has received notable attention from the scientific community (Peng et al., 2022). In addition to reducing the severity of the disorder by decreasing the apnea-hypopnea index (AHI) and the oxygen desaturation index (ODI), meta-analyses have highlighted the ability of exercise to improve several OSA-related outcomes, which include sleepiness, sleep quality, and sleep efficiency, as well as parameters like maximal oxygen consumption (VO_2_max), body mass index (BMI), and overall quality of life (Iftikhar et al., 2014; Aiello et al., 2016; Lins-Filho et al., 2020; Fank et al., 2022; Peng et al., 2022).

Despite these advances, the exact mechanisms underpinning the beneficial effects of exercise on symptom alleviation and OSA severity remain only partially understood (Torres-Castro et al., 2021). It is plausible that a single mechanism may not solely account for these effects; instead, a complex interplay of contributing factors could be responsible (Iftikhar et al., 2014). Notably, aerobic exercise interventions can potentially be used to manage obesity, a pivotal risk factor for developing OSA (Aiello et al., 2016). Additionally, resistance training interventions have shown the capacity to mitigate fluid accumulation in the legs, which might help prevent nocturnal fluid displacement toward the neck during sleep, thereby reducing the likelihood of upper airway collapse – an event implicated in OSA (Yumino et al., 2010; Mirrakhimov, 2013). Consequently, the combined implementation of these exercise modalities may not only promote synergistic benefits in OSA but also aligns with the health recommendations of the World Health Organization (Bull et al., 2020).

Although numerous studies have examined the impacts of exercise on OSA, adult populations have been the primary focus (Aiello et al., 2016; Lins-Filho et al., 2020; Peng et al., 2022). Thus, it is important to expand the scope of investigation by including older individuals who not only have a higher prevalence of OSA but are also more vulnerable to developing the disease (Edwards et al., 2010). It will therefore be possible to determine whether the beneficial effects of exercise observed in adults also apply to older adults.

The use of exercise as a tool for treating OSA may contribute to reducing healthcare costs. Exercise offers a low-cost option in terms of both implementation and monitoring and its cost-effectiveness has been demonstrated across different contexts (Roine et al., 2009; Garrett et al., 2011; Abu-Omar et al., 2017). Within the context of global healthcare spending, sleep disorders, including OSA, are the primary contributors (Mohit and Wickwire, 2020) and their prevalence continues to rise annually (Ensrud et al., 2020). Moreover, sleep-disordered breathing, which includes OSA, has substantial health, societal, and economic repercussions, thereby inflating healthcare expenses for both patients and society (Jennum and Kjellberg, 2011). These implications include vehicle and occupational accidents and different associated comorbidities (Knauert et al., 2015). It is therefore crucial to reduce the direct and indirect costs related to OSA, mainly through more cost-effective and noninvasive therapies (Wickwire et al., 2020).

Therefore, this study aims to investigate the effects of a combined exercise program with sleep education on sleep quality and on the severity of OSA in older adults. The results obtained will enable healthcare professionals to build their daily practice on evidence-based science, incorporating another non-pharmacological treatment option for older adults with OSA. The hypothesis is that the combination of resistance and aerobic exercises, coupled with sleep education, will be capable of reducing both the severity and symptoms of OSA in older adults when compared to the control group, which will exclusively receive educational recommendations for managing OSA.

## 2 Methods

### 2.1 Study design

**Figure 1:**
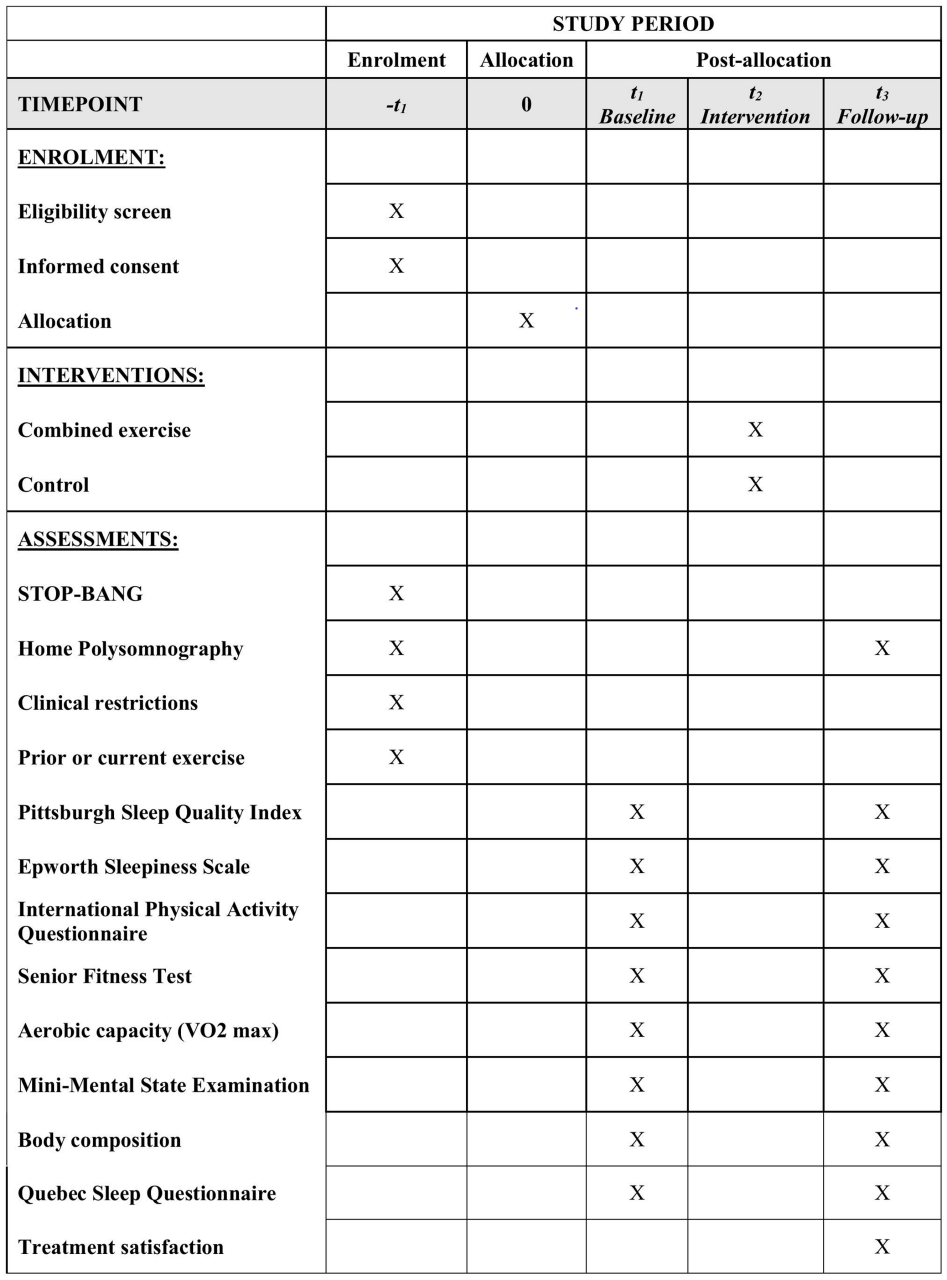
SPIRIT schedule of enrolment, interventions, and assessments.

This study is designed as a two-arm parallel group, randomized controlled trial that assesses the effects of a combined exercise program with sleep education on sleep quality and on the severity of OSA in older adults. Participants will be recruited from the waiting list for polysomnography in a city in southern Brazil. After initial screening, older people at high risk for OSA will undergo polysomnography. Older adults who meet the inclusion criteria will be randomized to the intervention or control group. The intervention will last 12 weeks, three times a week, and will be conducted at the Center for Health and Sports Sciences, State University of Santa Catarina, Brazil. The study was designed following the SPIRIT recommendations (Chan et al., 2013) and the schedule of enrolment, interventions, and assessments is shown in Figure 1.

### 2.2 Participants

**Figure 2:**
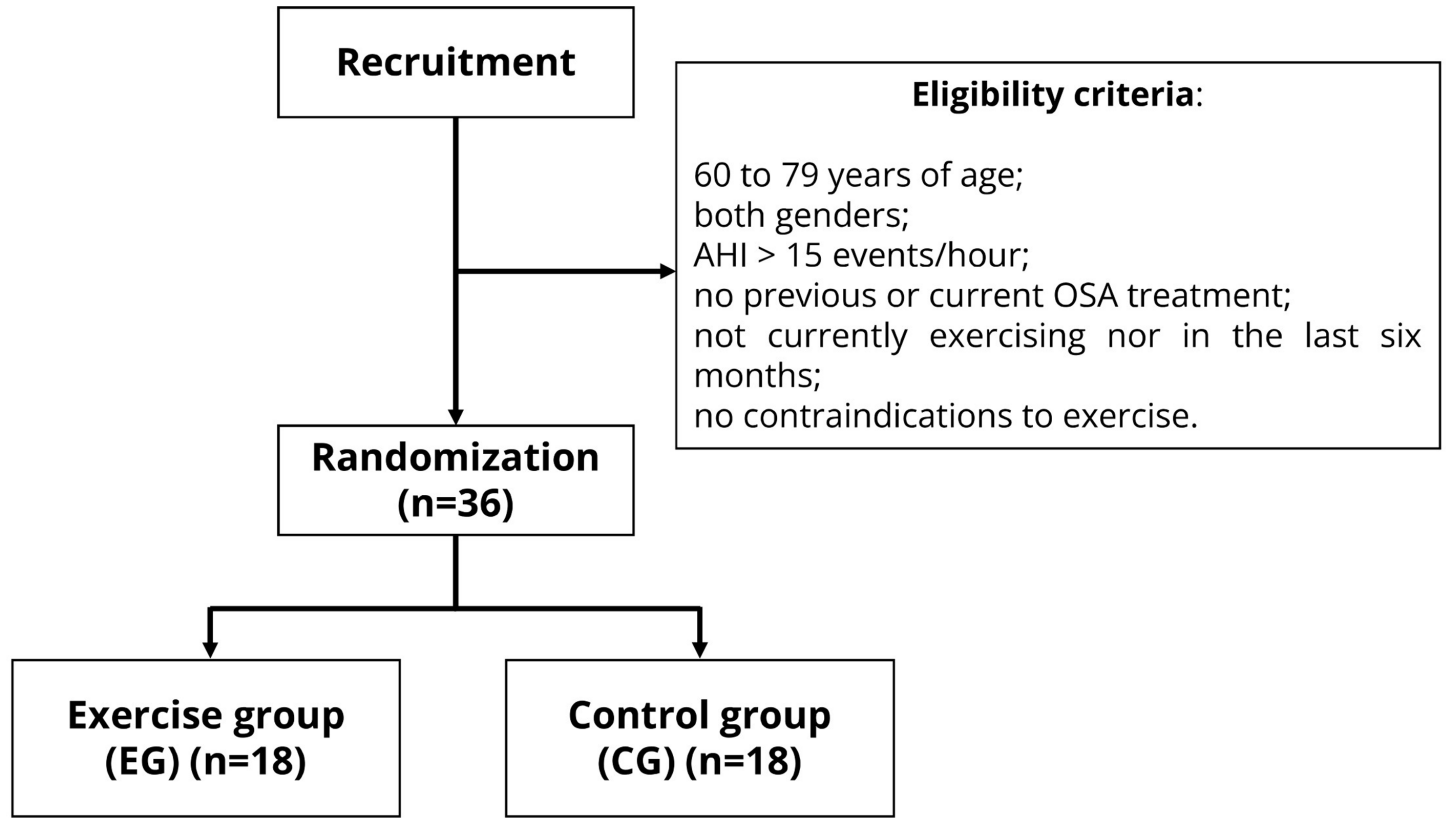
Recruitment and randomization of the clinical trial. AHI, apnea-hypopnea index; OSA, obstructive sleep apnea.

The following inclusion criteria will be applied to select the participants in this randomized clinical trial (Figure 2): age between 60 and 79 years; both genders; moderate to severe OSA with an AHI > 15 events per hour, and no history of previous or ongoing treatment for OSA. Older adults who have engaged in regular exercise in the last six months and those with contraindications (cardiovascular, respiratory, musculoskeletal, or neurological) to exercise will be excluded. Participants who do not adhere to the interventions at a rate ≥ 85% will also be excluded from the study.

The sample will be selected by non-probabilistic sampling. Participants will be recruited through partnerships with public and private institutions in Grande Florianopolis, Santa Catarina, Brazil. Additionally, the study will be promoted through healthcare services, elderly community groups, local media (radio and newspapers) and, if necessary, leafleting in neighborhoods near the university and during technical-scientific events.

### 2.3 Sample size

To determine the minimum number of participants required for the interventions, the sample size was calculated using the GPower 3.1 software. Using repeated measures ANOVA (within-subjects and between-subjects interaction) and assuming a significance level (α) of 0.05, power (β) of 0.95, and effect size of 0.35 (da Silva et al., 2022), the minimum sample size is 30 individuals. This number will be increased by 20% to account for potential sample losses, resulting in a minimum sample size of 36 participants divided into two groups: – the exercise group (EG) and the control group (CG), each consisting of 18 subjects. Losses after randomization will be included in the intention-to-treat analysis but not in the analysis of the protocol.

### 2.4 Eligibility outcomes

#### 2.4.1 Obstructive sleep apnea

To assess the risk of OSA in older adults, the STOP-BANG questionnaire (Chung et al., 2012) will be administered, which is designed to identify individuals at a higher risk of developing apnea. This screening instrument consists of eight questions that evaluate risk factors for OSA, with the responses being categorized as “yes” or “no.” The score ranges from 0 to 8 and scores ≥3 have shown high sensitivity in detecting moderate to severe OSA (Chung et al., 2012).

After the first assessment, participants classified as being at higher risk for OSA will undergo type III home polysomnography (HPSG) (Philips Respironics Alice NightOne). HPSG consists of the concurrent monitoring of sleep and respiration using thoracic and abdominal belts, an oximeter (to capture oxygen saturation and heart rate), and a nasal cannula (to monitor airflow and snoring), as well as the recording of body position during sleep (Jordan et al., 2014; Lee and Sundar, 2021). The following data will be obtained: AHI, ODI, minimum and mean oxyhemoglobin saturation (SaO2), sleep efficiency, sleep latency, and the type of respiratory event. Only individuals exhibiting an AHI ≥15 events per hour will be eligible for participation in the study.

#### 2.4.2 Clinical restrictions and prior or current regular exercise

Participants will be asked questions regarding their history of cardiovascular, respiratory, musculoskeletal, and neurological conditions to determine any clinical restrictions for participation in exercise. These questions developed by the research team are aimed at identifying potential health issues that may restrict the participant from participating in this clinical trial. The following two questions will be used to determine whether the participant currently exercises or has engaged in regular exercise in the last six months, which will also be used as inclusion criteria: (a) “Do you currently exercise?” and (b) “Have you regular exercise in the last six months?”

### 2.5 Primary outcomes

#### 2.5.1 Sleep quality

The Pittsburgh Sleep Quality Index (PSQI) (Buysse et al., 1989) will be used to evaluate sleep quality. The PSQI has been validated for use in Brazil (Bertolazi et al., 2011), showing high internal consistency (Cronbach’s α = 0.82). The questionnaire consists of 19 items that assess various sleep-related issues over the past month. The questions are divided into seven components and a score ranging from 0 to 3 is assigned to each component. These components include sleep quality, sleep duration, sleep latency, habitual sleep efficiency, sleep disturbances, medication use, and daytime dysfunction. The sum of scores for each component provides a global score ranging from 0 to 21, with higher scores reflecting poorer sleep quality. A score above 5 indicates poor quality (Buysse et al., 1989).

#### 2.5.2 Daytime sleepiness

Daytime sleepiness will be assessed with the Epworth Sleepiness Scale (ESS) (Johns, 1991). The instrument has been validated for use in Brazil, with a reliability coefficient of 0.83 (Bertolazi et al., 2009). The ESS is aimed at evaluating the likelihood of dozing off in eight situations known to induce sleepiness. Respondents are required to indicate the probability of falling asleep in each situation, which ranges from “would never doze” (0) to “high chance of dozing” (3). The overall score ranges from 0 to 24 and a score above 10 suggests a diagnosis of excessive daytime sleepiness (Johns, 1991).

### 2.6 Secondary outcomes

#### 2.6.1 Physical activity and sedentary behavior

The International Physical Activity Questionnaire (IPAQ) adapted for older adults will be used to investigate physical activity. This instrument has been validated for use in Brazilian older adults (Mazo and Benedetti, 2010), showing good reproducibility (Benedetti et al., 2004). The IPAQ estimates the weekly energy expenditure from physical activity across various domains. These domains include physical activity at work, physical activity during transportation, household physical activity, recreational, sports and exercise-related physical activity, and sedentary behavior.

The adapted version consists of 15 questions that evaluate moderate to vigorous physical activity; these activities should be performed for at least 10 consecutive minutes to be considered. In the current study, only sections four (recreational, sports and exercise-related physical activity) and five (sitting time) of the IPAQ (Mazo and Benedetti, 2010) will be administered and the time in minutes/week of moderate and/or vigorous physical activity will be calculated to classify the sample as active (≥150 min/week) or insufficiently active (<150 min/week).

#### 2.6.2 Physical fitness

The physical fitness of the participants will be assessed using the Senior Fitness Test battery (Rikli and Jones, 1999), which evaluates the physiological capacity of older adults to perform habitual activities of daily living. The battery consists of the following tests: chair stand to estimate lower limb strength; arm curl to assess upper limb strength; chair sit-and-reach to measure lower limb flexibility; back scratch to test upper limb flexibility; 8-foot up-and-go to analyze coordination, dynamic balance, and agility, and 6-min walk to evaluate aerobic endurance.

This battery will be used because of its specificity and validation in the elderly population (Rikli and Jones, 1999), its ease of application, and operational cost-effectiveness. In addition, this instrument has been used across different countries, including Brazil (Silva et al., 2019; Gonçalves et al., 2021). The results will be analyzed based on the difference (Δ) between the values obtained by the participants in the tests before and after the interventions.

#### 2.6.3 Aerobic capacity

Aerobic capacity will be assessed based on maximal oxygen consumption (VO_2_max). VO_2_max will be measured directly by an external researcher using a treadmill exercise test according to the modified Bruce protocol (Lerman et al., 1976). The test will be conducted using ergospirometry equipment capable of collecting direct measurements of O_2_ and CO_2_ concentrations, respiratory exchange ratio, heart rate, and ambient temperature and pressure in real-time. Pre-test instructions will be provided to the participants in accordance with the guidelines of the American Heart Association (Fletcher et al., 2013). For safety reasons, all tests will be supervised by a cardiology professional. Exhaustion will be defined when at least one of the following criteria is met: Borg rating of perceived exertion ≥18 out of 20 associated with a respiratory exchange ratio exceeding 1.10, and/or reaching a peak heart rate ≥ 85% of the predicted maximum heart rate, and/or achieving a point of stabilization in oxygen uptake (Vecchiato et al., 2022).

#### 2.6.4 Cognitive status

The Mini-Mental State Examination (MMSE) will be used to evaluate the cognitive status of the participants. The MMSE was developed by Folstein et al. (1975) and was validated for use in Brazil (Bertolucci et al., 1994). The instrument generates a maximum final score of 30 points, corresponding to high cognitive capacity. The cutoff values to determine preserved cognitive status take into account the educational level (Brucki et al., 2003).

#### 2.6.5 Body composition

Anthropometric and body composition measurements will be carried out to examine various outcomes. These measurements will be used to determine possible correlations with changes in the primary outcomes of OSA after the intervention. Body weight will be measured with a G-Life^®^ Millenium digital scale (silver model CA6000). Height will be measured with a Cardiomed^®^ stadiometer (height limit of 2.16 m) from the feet to the highest point of the head. The body mass index (BMI) will be calculated from the body weight and height using the formula: BMI (kg/m^2^) = body weight (kg)/height^2^ (m). Neck circumference (NC) will be measured with a Cescorf^®^ measuring tape immediately above the thyroid cartilage.

Body composition will also be evaluated by whole-body dual-energy X-ray absorptiometry (DXA) using a Hologic system (Discovery Wi Fan-Beam, Bedford, Massachusetts, United States). The equipment will be calibrated daily and weekly according to the manufacturer’s recommendations. The same lab technician will position the participant for each scan, perform the scans, and analyze the data according to the operator’s manual using the standard analysis protocol. DXA provides data on bone mineral content (g), bone mineral density (g/cm^2^), lean mass (kg), and fat mass (kg).

#### 2.6.6 Quality of life

Quality of life will be analyzed using the Quebec Sleep Questionnaire (Lacasse et al., 2004), an instrument specifically designed to assess the quality of life of individuals with OSA. The questionnaire has been translated and adapted for use in Brazil (de Melo et al., 2017) and comprises 32 questions that evaluate the impact of apnea across five distinct domains: daytime sleepiness, daytime symptoms, nighttime symptoms, emotions, and social interactions. Each domain consists of four to 10 items, each scored on a Likert scale ranging from 1 to 7. The results will be presented individually for each domain considering the mean score obtained for each domain (Lacasse et al., 2004).

#### 2.6.7 Treatment satisfaction

To evaluate satisfaction with the treatments, participants will complete a brief questionnaire based on the study by Kline et al. (2011), which assesses the expected changes in the severity of OSA, sleep quality, daytime sleepiness, mood, and overall health. Responses will be rated on a Likert scale, where 1 = much worse and 5 = much better. Additionally, participants will be asked about their overall treatment satisfaction using a Likert scale, where 1 = very dissatisfied and 2 = very satisfied.

### 2.7 Data collection

Before the recruitment process, participants will receive an explanation of the objectives and assessment procedures of the study. Upon agreeing to participate, the older adults will read and sign the informed consent form. Participants will then respond to the questionnaires and undergo HPSG to determine their eligibility for the study. After confirmation of their eligibility, older adults will complete the remaining questionnaires and undergo the remaining tests. Assessments regarding HPSG will be conducted by an assessor blind to treatment allocation. Because of the inherent characteristics of the intervention, it is not possible to maintain blinding for either the participants or the researcher regarding allocation. However, both are strongly advised against revealing the allocation status of participants during follow-up assessments.

**Figure 3:**
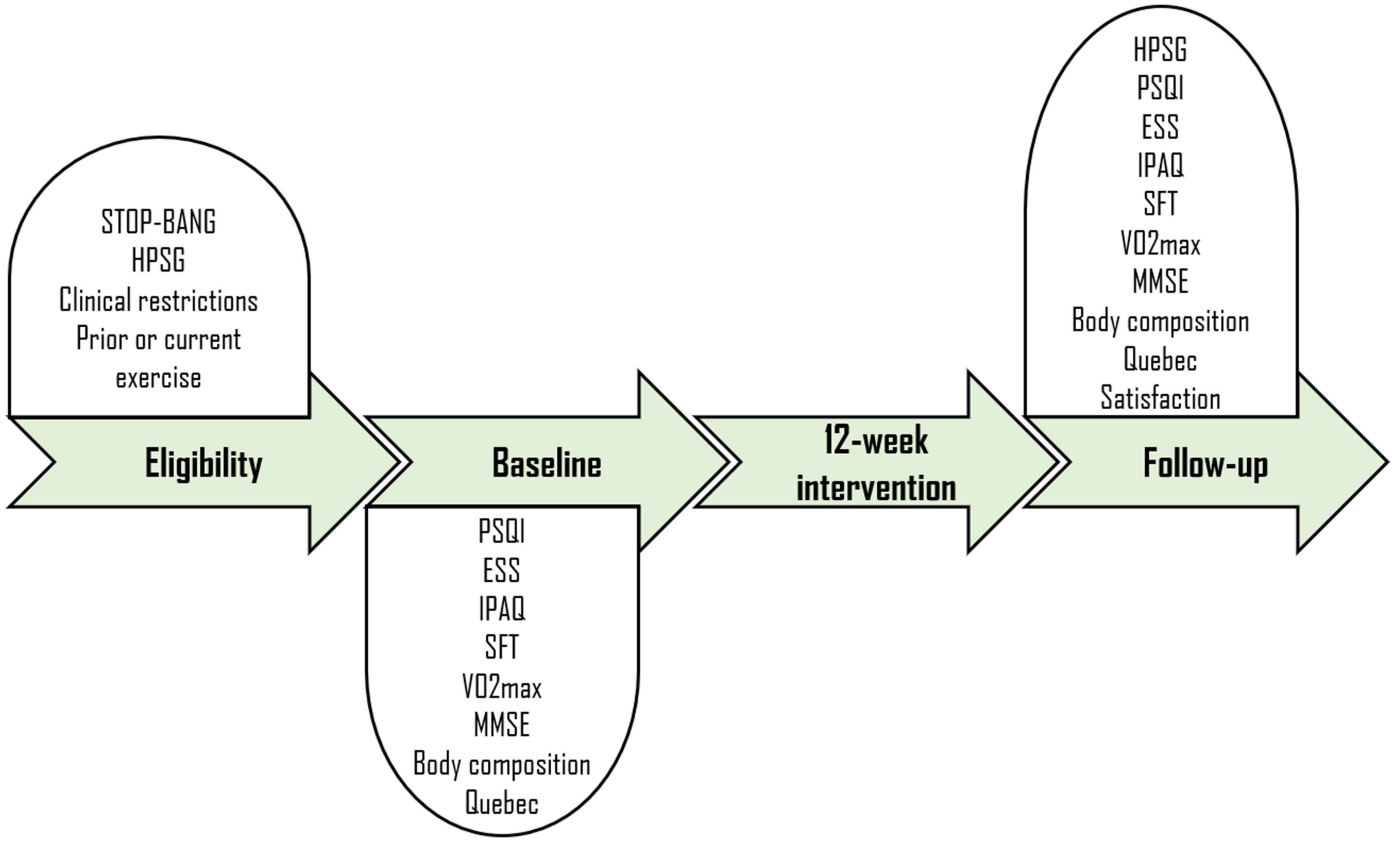
Clinical trial design. HPSG, Home Polysomnography; PSQI, Pittsburgh Sleep Quality Index; ESS, Epworth Sleepiness Scale; IPAQ, International Physical Activity Questionnaire; SFT, Senior Fitness Test; MMSE, Mini-Mental State Examination.

Next, participants will be randomized to two groups: the exercise group (EG) and the control group (CG). To ensure similarity in participant characteristics within the groups, stratified randomization will be conducted to control the covariates sex (2 levels: male and female) and OSA severity (2 levels: moderate and severe). For this purpose, an external researcher will generate a computerized sequence of random numbers to determine the 1:1 allocation to either EG or CG based on the order of participant recruitment. The specific schedules for instrument administration and the data collection procedure are outlined in Figure 3.

### 2.8 Experimental design

As previously mentioned, participants will be allocated to either EG or CG. Participants randomized to EG will undergo a 12-week intervention, three times per week, totaling 36 sessions. Each training session will last approximately 60 min and will be organized as follows: warm-up (5 min), training (50–60 min), and cool-down (5 min). Training intensities will be progressively adjusted throughout the weeks according to the principle of overload.

The intervention will consist of a training program that combines aerobic and resistance exercises within the same session. This approach is based on other studies that have applied similar training programs to adults with OSA (Kline et al., 2011; Desplan et al., 2014; Bughin et al., 2020). The combined exercise approach is also in line with the physical activity recommendations for Brazilian older adults, which suggest the prescription of both types of exercise (de Coelho-Ravagnani et al., 2021). Each training session will start with a warm-up (5 min), followed by aerobic exercise (20 min) and resistance exercises (30 to 40 min), and concluding with a cool-down (5 min).

The aerobic training protocol will be conducted on a treadmill or stationary bicycle, with intensity corresponding to the anaerobic threshold determined based on the ventilatory thresholds established in the stress test. Pre-test instructions will be provided to participants in accordance with the recommendations of the American Heart Association (Fletcher et al., 2013). Heart rate will be monitored during the sessions using a Polar heart rate monitor.

The resistance exercise protocol will consist of exercises for the legs, arms, chest, back, and abdomen. All exercises will comprise three sets, with a rest interval of 90 s. Additionally, the intervention will be divided into three phases: familiarization, load determination, and training. The familiarization phase will involve learning the proper range of motion, breathing, and rhythm for each exercise. Participants will be instructed to inhale during the eccentric phase and to exhale during the concentric phase of the exercise, performing repetitions at a tempo of two seconds for each phase of the movement.

Loads will be determined using the maximum repetition (RM) test to define loads within the training target zone, which will be set at 10 RM in this study. This load determination is based on various studies (Jambassi Filho et al., 2011; Gurjão et al., 2012; Virtuoso et al., 2019). Participants will be instructed to perform as many repetitions as possible with a load subjectively chosen by the researcher (trial and error). During this determination process, a maximum of two attempts will be made for load definition, with a recovery interval of 3 to 5 min. If a participant completes more than 12 repetitions, 1 kg will be added for every two additional repetitions. If the number of repetitions falls below 8 RM, the load will be adjusted through trial and error. To ensure accuracy in load definition and to assess reproducibility, the tests will be repeated after a minimum interval of 48 h. Following the load determination, participants will start the resistance exercises as part of the intervention.

In addition to the exercise, participants in EG will also receive sleep education, which will include information on sleep hygiene and on the pathophysiology, risk factors, and treatments of OSA. Sleep education will also be administered to participants randomized to CG so that they would adhere to the recommendations of the American Academy of Sleep Medicine (AASM) (Epstein et al., 2009). A booklet containing information about sleep hygiene will be provided to CG participants before the start of the intervention. Furthermore, recorded videos on the pathophysiology, risk factors, and treatments for OSA will be sent remotely to the participants. These materials will be systematically distributed over the 12-week period. The participants will also be asked to maintain their normal activities and not to perform exercise. After the research period, CG participants will be referred for appropriate treatment according to medical recommendation.

### 2.9 Ethical standards

The ethical principles of the Declaration of Helsinki will be followed. The research proposal was formally submitted to the Ethics Committee for Research Involving Humans of the State University of Santa Catarina and received approval under number 5.729.066 (CAAE: 60046022.5.0000.0118). Additionally, the clinical trial was submitted and approved (RBR-9hk6pgz) by the Brazilian Registry of Clinical Trials (ReBEC). Prospective subjects who meet the eligibility criteria will be invited to participate in the study. Upon agreement, participants will start the interventions only after they had thoroughly read and signed the informed consent form.

### 2.10 Data analysis

The data will be organized using Microsoft Excel and subjected to statistical analysis using the IBM SPSS 20.0 software. Descriptive statistics will be employed to assess all variables and will include absolute frequency, relative frequency, mean, and standard deviation. Data distribution normality will be tested by the Shapiro–Wilk test. Baseline characteristics will be examined based on the nature of the variables (numerical or categorical). Differences between groups at baseline will be evaluated using one-way analysis of variance (ANOVA) or the Kruskal-Wallis test for numerical variables and Pearson’s chi-square or Fisher’s exact test for categorical variables.

Repeated-measures ANOVA will be applied to assess post-intervention group-by-time interactions. Multiple comparisons will be performed using Tukey’s post-hoc test. Furthermore, potential associations between deltas (Δ) – mean changes before and after the intervention – of OSA-related outcomes (AHI and ODI) and possible explanatory variables (VO_2_, BMI, and NC) will be explored using Pearson’s or Spearman’s correlation test, depending on data normality. A 95% confidence interval will be employed in all analyses, corresponding to a significance level of 0.05 (5%). As highlighted previously, losses after randomization will be included in the intention-to-treat analysis but not in the analysis of the protocol. (Supplementary Table S1) shows the summary of the outcomes along with their respective measurement tool.

## 3 Discussion

This study aims to investigate the impact of a combined exercise program and sleep education on the severity and symptoms associated with OSA among older adults. The hypothesis posits that the combination of resistance and aerobic exercises, along with sleep education, will effectively reduce both the severity and the symptoms of OSA in older adults compared to the control group. This protocol is based on previous research demonstrating the potential of combined physical exercise to mitigate OSA severity in middle-aged adults (Kline et al., 2011; Desplan et al., 2014; Bughin et al., 2020).

The strengths of this study include the direct assessment of OSA using HPSG and the analysis of various symptoms associated with the disorder such as sleep quality and efficiency, as well as the measurement of anthropometric variables. The use of validated instruments increases the validity and reliability of the results. Participant recruitment will be broad but specific, with the inclusion criteria being based on previous studies and considering the specificity of OSA. Furthermore, the ecological validity of the study is considered, especially in relation to the exercise intervention, enabling its application in clinical practice. These strengths provide a solid framework and will thus increase the internal validity and clinical relevance of the study, which will contribute to scientific knowledge and improve the quality of life of older people with OSA.

The present study is of great importance for the scientific advancement in this field since it aims to investigate whether the positive effects of combined exercise on OSA are also observed in older people. If the study results demonstrate significant improvements in sleep parameters and OSA severity after the combined exercise program, this may indicate an effective non-pharmacological intervention for treating these conditions in older adults. This discovery would be of great clinical relevance by adding a non-pharmacological therapeutic option to the treatment of OSA in this population, which could be used as an adjunct to existing approaches. Moreover, effective intervention for managing sleep apnea in older adults may result in substantial savings in public health costs by preventing complications caused by OSA and reducing the need for expensive medical treatments.

Furthermore, the potential results may equip healthcare professionals with empirical data for their daily practice, enabling the incorporation of evidence-based strategies in the non-pharmacological treatment modalities for OSA. This approach has the potential to improve the quality of life of older people with OSA; in addition, it provides a safer and more affordable alternative to pharmacological interventions. This study also has the potential to boost initiatives that promote physical exercise among older adults as an essential part of geriatric care. Therefore, the findings of this study may have important impacts both in the field of sleep research and in clinical practice, providing an additional scientifically supported option for the treatment of OSA in older adults.

## Ethics statement

The studies involving humans were approved by the Ethics Committee for Research Involving Humans of the State University of Santa Catarina. The studies were conducted in accordance with the local legislation and institutional requirements. The participants provided their written informed consent to participate in this study.

## Author contributions

FF: Conceptualization, Methodology, Writing – original draft, Writing – review & editing. RA: Methodology, Writing – review & editing. MS: Methodology, Writing – review & editing. AE: Methodology, Writing – review & editing. DM: Methodology, Supervision, Writing – review & editing. GM: Conceptualization, Methodology, Supervision, Writing – review & editing.
