# Supplementary material for: Effects of combined exercise training with sleep education in older adults with obstructive sleep apnea: protocol for a randomized clinical trial
Source: Front Psychol. 2024 Feb 15;15:1322545. doi: 10.3389/fpsyg.2024.1322545 (PMC10902716; doi:10.3389/fpsyg.2024.1322545)
Supplement: Supplementary file 1 [file Table_1.docx]

**Supplementary material**

Table S1. Summary of the outcomes along with their respective measurement tool.

| **Eligibility outcomes** | **Measurement tools** |
| --- | --- |
| Risk of Obstructive Sleep Apnea | STOP-BANG |
| Apnea-hypopnea index | Home polysomnography |
| Clinical restrictions and prior or current regular exercise | Questions developed by the research team |
| **Primary outcomes** |  |
| Sleep quality | Pittsburgh Sleep Quality Index |
| Daytime sleepiness | Epworth Sleepiness Scale |
| **Secondary outcomes** |  |
| Physical activity and sedentary behavior | International Physical Activity Questionnaire |
| Physical fitness | Senior Fitness Test |
| Aerobic capacity | Maximal oxygen consumption |
| Cognitive status | Mini-Mental State Examination |
| Body composition | Whole-body dual-energy X-ray absorptiometry |
| Quality of life | Quebec Sleep Questionnaire |
| Treatment satisfaction | Questionnaire |
